# Etiology of diminished left ventricular function in patients with Tetralogy of Fallot by cardiac magnetic resonance

**DOI:** 10.1186/1532-429X-11-S1-P173

**Published:** 2009-01-28

**Authors:** David HF Gommans, Maureen J Van der Vlugt, Jeroen W Op den Akker, Erwin S Zegers, Arie PJ Van Dijk

**Affiliations:** 1 Heartlung Centre, Radboud University Medical Centre Nijmegen, Nijmegen, Netherlands; 2 Department of Radiology, Radboud University Medical Centre Nijmegen, Nijmegen, Netherlands

**Keywords:** Right Ventricular, Cardiac Magnetic Resonance, Left Ventricular Function, Right Ventricular Function, Left Ventricular Cavity

## Introduction

Right ventricular (RV) function is impaired in patients with Tetralogy of Fallot due to right bundle branch block and volume overload by pulmonary regurgitation. As a consequence, paradoxical septal motion develops. Daily practice suggests left ventricular (LV) function is also reduced. Paradoxical septal motion could be one of the mechanisms causing diminished LV function.

## Purpose

The purpose of this CMR study was to measure paradoxical septal motion and its effect on LV function in patients with Tetralogy of Fallot.

## Methods

Twenty nine patients (17 males) with Tetralogy of Fallot with a mean age of 29 ± 10 years were included. MR imaging was performed on Siemens Avanto 1.5 Tesla scanner. Data-analysis was performed with the MR Analytical Software System (Medis, Leiden, The Netherlands) to measure end-diastolic (EDV) and end-systolic (ESV) volumes and calculate ejection fraction (EF) for both ventricles. Paradoxical septal motion was quantified in end-diastole by the curvature of the septum, defined as 1 divided by the radius of the curvature and a negative value corresponds to septal motion towards the LV cavity. MR phase-contrast flow quantification was performed to assess pulmonary regurgitation. ECG analysis for QRS duration was performed to diagnose right bundle branch block. Pearson correlation tests were done to assess correlations between variables.

## Results

RV EDV and ESV were 227.0 ± 53.3 and 148.4 ± 47.7 ml, respectively. LV EDV and ESV were 119.4 ± 24.5 and 55.9 ± 17.9 ml, respectively. RV function was reduced (EF = 35.3 ± 10.2%) and LV function was also mildly reduced (EF = 53.5 ± 9.5%). All patients had reduced curvature (0.14 ± 0.08), but in only 1 case the septum moved towards the LV cavity. Mean pulmonary regurgitation fraction was 43.6 ± 17.6%. Mean QRS duration was 144 ± 30 msec. Significant correlations were found between curvature and pulmonary regurgitation fraction (r = 0.39, p < 0.05), pulmonary regurgitation fraction and RV EDV (r = 0.49, p < 0.01), RV ESV volumes and QRS duration (r = 0.56, p < 0.01) and between QRS duration and RVEF (r = -0.676, p < 0.01). No significant correlations could be found concerning LV function.

## Conclusion

Patients with Tetralogy of Fallot have dilated right ventricles with impaired function due to pulmonary regurgitation and right bundle branch block leading to paradoxical septal motion. Paradoxical septal motion was seen in all patients with Tetralogy of Fallot. Although in this population with only mildly reduced LV function and mild paradoxical septal motion a correlation between both could not be demonstrated, further research in a more heterogeneous population is warranted.

